# Integrating Histologic and Genomic Characteristics to Predict Tumor Mutation Burden of Early-Stage Non-Small-Cell Lung Cancer

**DOI:** 10.3389/fonc.2020.608989

**Published:** 2021-04-30

**Authors:** Yuan Qiu, Liping Liu, Haihong Yang, Hanzhang Chen, Qiuhua Deng, Dakai Xiao, Yongping Lin, Changbin Zhu, Weiwei Li, Di Shao, Wenxi Jiang, Kui Wu, Jianxing He

**Affiliations:** ^1^ National Clinical Research Center of Respiratory Disease, The First Affiliated Hospital of Guangzhou Medical University, Guangzhou, China; ^2^ State Key Laboratory of Respiratory Diseases, The First Affiliated Hospital of Guangzhou Medical University, Guangzhou, China; ^3^ The Translational Medicine Laboratory, The First Affiliated Hospital of Guangzhou Medical University, Guangzhou, China; ^4^ BGI Genomics, BGI-Shenzhen, Shenzhen, China; ^5^ BGI-Shenzhen, Shenzhen, China; ^6^ China National GeneBank, BGI-Shenzhen, Shenzhen, China

**Keywords:** early-stage non-small-cell lung cancer, tumor mutation burden (TMB), histology, genomics, model

## Abstract

Tumor mutation burden (TMB) serves as an effective biomarker predicting efficacy of mono-immunotherapy for non-small cell lung cancer (NSCLC). Establishing a precise TMB predicting model is essential to select which populations are likely to respond to immunotherapy or prognosis and to maximize the benefits of treatment. In this study, available Formalin-fixed paraffin embedded tumor tissues were collected from 499 patients with NSCLC. Targeted sequencing of 636 cancer related genes was performed, and TMB was calculated. Distribution of TMB was significantly (p < 0.001) correlated with sex, clinical features (pathological/histological subtype, pathological stage, lymph node metastasis, and lympho-vascular invasion). It was also significantly (p < 0.001) associated with mutations in genes like *TP53*, *EGFR*, *PIK3CA*, *KRAS*, *EPHA3*, *TSHZ3*, *FAT3*, *NAV3*, *KEAP1*, *NFE2L2*, *PTPRD*, *LRRK2*, *STK11*, *NF1*, *KMT2D*, and *GRIN2A*. No significant correlations were found between TMB and age, neuro-invasion (p = 0.125), and tumor location (p = 0.696). Patients with *KRAS* p.G12 mutations and *FAT3* missense mutations were associated (p < 0.001) with TMB. *TP53* mutations also influence TMB distribution (P < 0.001). TMB was reversely related to *EGFR* mutations (P < 0.001) but did not differ by mutation types. According to multivariate logistic regression model, genomic parameters could effectively construct model predicting TMB, which may be improved by introducing clinical information. Our study demonstrates that genomic together with clinical features yielded a better reliable model predicting TMB-high status. A simplified model consisting of less than 20 genes and couples of clinical parameters were sought to be useful to provide TMB status with less cost and waiting time.

## Introduction

Immune checkpoint inhibitors (ICIs) targeting programmed death 1 (PD-1) and programmed death ligand 1 (PD-L1) achieved great success improving clinical outcomes of patients with advanced NSCLC. The efficacy of ICIs varies widely among individuals (1). Therefore, biomarkers stratifying patients who may benefit from ICI treatment are of great importance. Immunostaining of PD-L1 is considered as the first considered option. NSCLC patients with tumor proportion score (TPS) ≥1% showed survival advantage from ICIs, especially, mono-immunotherapy. Tumor mutation burden (TMB) has been confirmed as a biomarker associating with efficacy of immunotherapy (2, 3). Meanwhile, in patients with resected NSCLC, TMB can help to evaluate long-term prognosis (4). Recent studies have shown that there are many factors affecting TMB distribution and ICIs (5, 6). TMB was negatively associated with clinical outcomes in metastatic *EGFR* mutant lung cancer patients treated with *EGFR*-TKI (7). *PIK3CA* amplification was significantly associated with TMB-H (8). Thus MSI-H/MMR-deficient tumors have much more somatic-mutations than MSS/MMR-proficient tumors (9), which have been demonstrated to have direct effects on TMB. Moreover, the molecular profile was associated with clinicopathological features and genetic ancestry markers of CRC patients (10). NSCLC tumors with elevated TMB and PD-L1 expression are associated with lympho-vascular invasion (11). It was also reported in patients with advanced gastric cancer that clinicopathological (lymph node metastasis) and molecular characteristics (*PIK3CA* mutations) are associated with responders to nivolumab (12).

TMB was precisely evaluated by whole-exon sequencing and could be predicted by a comprehensive genomic profiling (CGP) panel with a minimal size of 1 M. However, more turn-around time (TAT) would be taken when CGP is performed. Therefore, establishing a precise TMB predicting model is essential to monitor which populations are likely to respond to immunotherapy or prognosis and to maximize the benefits of treatment. In this article, we firstly aimed to select potential parameters by associating genetic and pathological characters with TMB distribution. An optimal TMB prediction model was constructed based on selected various clinical and genetic factors. Receiver operating curve analysis was applied to assess the performance of this prediction model.

## Materials and Methods

### Patients

A total of 499 Formalin-Fixed, Paraffin Embedded tumor specimens of resected lung cancer were collected between March 2019 and September 2019. All patients signed the informed consent. Five hundred and eight cancer-related genes were sequenced.

### Targeted Exome Capture Sequencing and Tumor Mutation Burden Assessment

Targeted exome capture sequencing data from 499 NSCLC samples were generated by MGI-500 platform. In detail, genomic DNA (gDNA) was extracted from FFPE and peripheral blood samples using the Qiagen DNeasy Blood & Tissue Kit (Qiagen, Hilden, Germany) per protocol. DNA concentration and quality were assessed by Qubit (Life Technologies, Gaithersburg, MD, USA) and agarose gel electrophoresis. gDNA (250 ng) was used for sequencing library construction as previously described. The hybridization product was subsequently purified, amplified, and qualified. Finally, sequencing of 508 key cancer related genes was performed with a paired-end 100 bp and 8 bp barcode on a MGISEQ-2000 sequencer following the manufacturer’s protocols.

Raw data was first filtered by SOAPnuke to exclude reads with low quality. The clean reads were then aligned to the reference human genome (UCSC hg19) using the BWA MEM algorithm. Single-nucleotide variants (SNVs) were detected by Genome Analysis Toolkit (GATK) Unified Genotyper. Small insertions and deletions (indels) were called using GATK Haplotype. Copy number variants (CNVs) were called using read-depth analysis. All the above variants were further filtered by quality depth, strand bias, mapping quality, and read position. Each variant was finally annotated with respect to gene location.

Targeted exome capture sequencing data of 499 NSCLC patients was analyzed in depth, and TMB was evaluated, which was defined as the total number of non-synonymous and indel somatic mutations present in a baseline tumor sample excluding known driver genes. TMB-high group was defined based on top 20% of TMB value.

### Statistical Analysis

Chi-square test and Kruskal–Wallis test were used for comparing categorical and continuous variables, respectively. A P-value threshold of p ≤0.001 (Chi-square test) and p <0.05 (Kruskal–Wallis test) were used to define statistical significance. To determine the driver genes’ differential between TMB-H and TMB-L, the Wilcoxon test was performed to figure out the significant driver genes. To determine the multivariable association of clinical and mutation characteristics with TMB-H, LASSO regression was used. In order to evaluate whether the histologic and genomic data could provide effective prediction of TMB, the receiver operating characteristic (ROC) curve analysis and area under the curve (AUC) were applied to evaluate the accuracy of TMB prediction model.

## Results

### Sample Demographics and Clinical Characteristics

**Figure 1:**
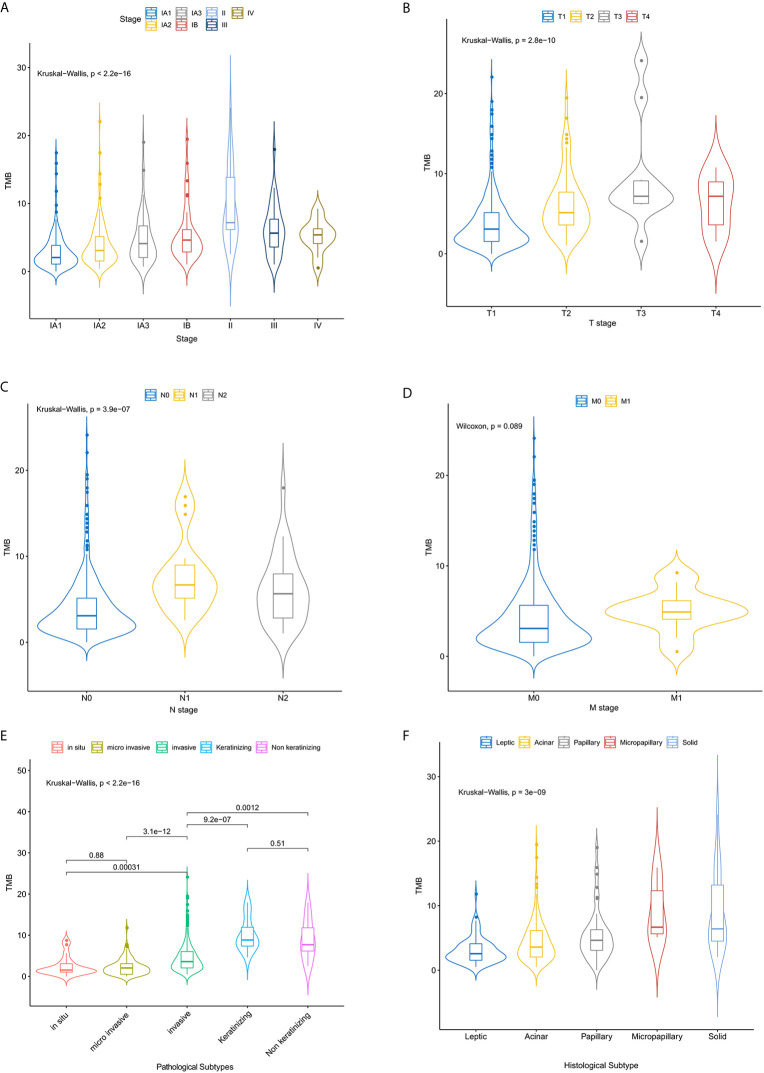
The relationship between the TMB distribution and the tumor stages of NSCLC patients. Correlation analysis of TMB and TNM stage **(A–D)**, pathological **(E)** and histological subtypes **(F)**.

499 FFPE tissues were collected from patients with clinically diagnosed NSCLC, including 470 lung adenocarcinomas (LUADs) and 29 lung squamous cell carcinomas (LUSCs). Fifty-five percent of the samples were female (n = 275) (**Table 1**). The median age at diagnosis was 60 years (range, 29 to 85 years). More males than females were found in the TMB-high group (P < 0.001) (**Table 1**). More patients with LUSC were in the TMB-high group compared with those with LUAD (P < 0.001) (**Table 1**). 216 samples were located in the left lung, and the other 283 samples were in the right lung. In the left lung, 69 (13.9%) cases were located in the lower left lobe, while 29.1% of NSCLC were located in the upper lobe. The distribution of TMB is not significantly affected by tumor location (**Table 1**). In this study, most patients were stage IA (n = 360; 72.0%) and IB (n = 44; 8.9%). Seventy-three (14.4%) patients belonged to stage II/III/IV (7.6%). Distribution of T stage was as follows: T1 (n = 388, 81.3%), T2 (n = 72, 15.1%), T3 (n = 10, 2.1%), and T4 (n = 7, 1.5%). Most of them were N0 (n = 440, 89.8%) and M0 stage (n = 483, 97.6%) (**Table 1**). Clinical stage (IA), T1, and N0 are significantly related to higher level of TMB (**Table 1** and **Figures 1A–D**). Here, we also find a significant association between TMB distribution and clinicopathological features such as pathological subtype (P < 0.001) (**Figure 1E**), histological subtype (P < 0.001) (**Figure 1F**), para-bronchial lymph nodes (P < 0.001), lymph node metastasis (P = 0.009), and LVI (lympho-vascular invasion) (P < 0.001) (**Table 1**). But, TMB distribution is not significantly affected by neuro-invasive (p = 0.125) (**Table 1**).

**Table 1:** Baseline characteristics of included patients by TMB.

	TMB low (N = 400)	TMB high (N = 99)	Total (N = 499)	p value
**Gender**				<0.001
Female	251 (62.8%)	24 (24.2%)	275 (55.1%)	
Male	149 (37.2%)	75 (75.8%)	224 (44.9%)	
**Age**				0.003
<= 60	220 (55.0%)	38 (38.4%)	258 (51.7%)	
>60	180 (45.0%)	61 (61.6%)	241 (48.3%)	
**Location**				0.627
Left	171 (42.8%)	45 (45.5%)	216 (43.3%)	
Right	229 (57.2%)	54 (54.5%)	283 (56.7%)	
**Histology**				<0.001
N-Miss	2	0	2	
LUAD	391 (98.2%)	77 (77.8%)	468 (94.2%)	
LUSC	7 (1.8%)	22 (22.2%)	29 (5.8%)	
**Pathological Subtype**				<0.001
N-Miss	10	1	11	
*In situ*	18 (4.6%)	2 (2.0%)	20 (4.1%)	
invasive	279 (71.5%)	71 (72.4%)	350 (71.7%)	
Keratinizing	1 (0.3%)	13 (13.3%)	14 (2.9%)	
Micro-invasive	89 (22.8%)	4 (4.1%)	93 (19.1%)	
Non-keratinizing	3 (0.8%)	8 (8.2%)	11 (2.3%)	
**Histological subtype**				<0.001
N-Miss	115	31	146	
Leptic	87 (30.5%)	8 (11.8%)	95 (26.9%)	
Acinar	136 (47.7%)	33 (48.5%)	169 (47.9%)	
Papillary	36 (12.6%)	12 (17.6%)	48 (13.6%)	
Micropapillary	2 (0.7%)	3 (4.4%)	5 (1.4%)	
Solid	9 (3.2%)	9 (13.2%)	18 (5.1%)	
Mucinous	15 (5.3%)	3 (4.4%)	18 (5.1%)	
**Lymph node**				0.009
N-Miss	214	40	254	
Negative	171 (91.9%)	47 (79.7%)	218 (89.0%)	
Positive	15 (8.1%)	12 (20.3%)	27 (11.0%)	
**Para-bronchial**				<0.001
N-Miss	201	36	237	
Negative	188 (94.5%)	50 (79.4%)	238 (90.8%)	
Positive	11 (5.5%)	13 (20.6%)	24 (9.2%)	
**LVI**				0.001
N-Miss	100	15	115	
0	264 (88.0%)	62 (73.8%)	326 (84.9%)	
1	36 (12.0%)	22 (26.2%)	58 (15.1%)	
**Stage**				<0.001
IA1	165 (41.2%)	14 (14.1%)	179 (35.9%)	
IA2	112 (28.0%)	21 (21.2%)	133 (26.7%)	
IA3	50 (12.5%)	18 (18.2%)	68 (13.6%)	
IB	34 (8.5%)	11 (11.1%)	45 (9.0%)	
II	10 (2.5%)	19 (19.2%)	29 (5.8%)	
III	20 (5.0%)	13 (13.1%)	33 (6.6%)	
IV	9 (2.2%)	3 (3.0%)	12 (2.4%)	
**T stage**				<0.001
N-Miss	21	1	22	
T1	329 (86.8%)	59 (60.2%)	388 (81.3%)	
T2	44 (11.6%)	28 (28.6%)	72 (15.1%)	
T3	3 (0.8%)	7 (7.1%)	10 (2.1%)	
T4	3 (0.8%)	4 (4.1%)	7 (1.5%)	
**N stage**				<0.001
N-Miss	7	2	9	
N0	365 (92.9%)	75 (77.3%)	440 (89.8%)	
N1	8 (2.0%)	11 (11.3%)	19 (3.9%)	
N2	20 (5.1%)	11 (11.3%)	31 (6.3%)	
**M stage**				0.783
N-Miss	3	1	4	
M0	387 (97.5%)	96 (98.0%)	483 (97.6%)	
M1	10 (2.5%)	2 (2.0%)	12 (2.4%)	

### Mutation Burden and Frequently Mutated Genes

**Figure 2:**
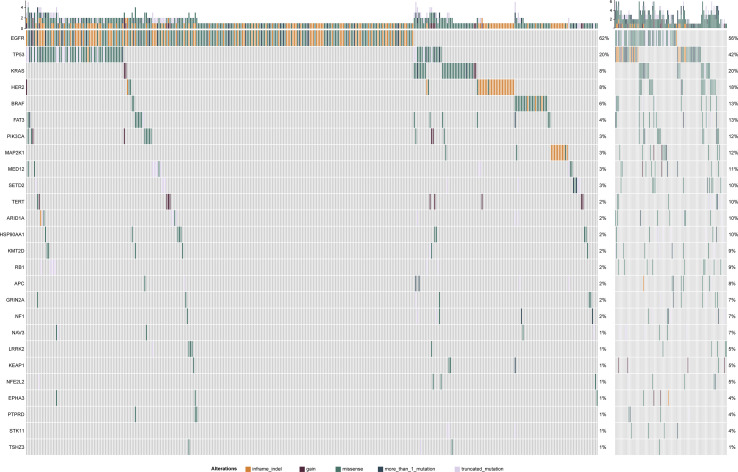
The left panel is TMB-L mutation map and the right panel is TMB-H mutation map. Mutation ratio of different genes displays in left. Different mutation types have different color codes.

Samples were divided into high (99) and low (400) TMB groups (**Table 1**) according to TMB top 20% in all histology (n = 6.15/Mb) (**Supplementary Figure 1**). In two groups, canonical driver mutations were found in *EGFR* (TMB-L: n = 246, TMB-H: n = 42), *KRAS* (TMB-L: n = 32, TMB-H: n = 18), *PIK3CA* (TMB-L: n = 13, TMB-H: n = 12), *BRAF* (TMB-L: n = 23, TMB-H: n = 5) and *TP53* (TMB-L: n = 80, TMB-H: n = 55) (**Supplementary Table 1** and **Figure 1**). There is no association between TMB distribution and driver gene mutational status (P = 0.27) (**Supplementary Figure 2**). Genes, differentially mutated between TMB-L and TMB-H patients (TMB-Low *vs*. TMB-High) were *EGFR* (62 *vs* 42%, P < 0.001), *EPHA3* (2 *vs* 13%, P < 0.001), *FAT3* (4 *vs* 20%, P < 0.001), *KEAP1* (1 *vs* 7%, P = 0.001), *KMT2D* (2 *vs* 10%, P < 0.001), *LRRK2* (1 *vs* 10%, P < 0.001), *NAV3* (1 *vs* 12%, P < 0.001), *NF1* (2 *vs* 10%, P < 0.001), *NFE2L2* (1 *vs* 11%, P < 0.001), *PIK3CA* (3.2 *vs* 12.1%, P < 0.001), *PTPRD* (1 *vs* 10%, P < 0.001), *STK11* (1 *vs* 8%, P < 0.001), *TP53* (20 *vs* 56%, P < 0.001), and *TSHZ3* (1 *vs* 13%, P < 0.001) (**Figure 2**, **Supplementary Table 1** and **Supplementary Figure 3**). At the same time, some gene mutations associated with immunotherapy resistance are not related to genetic mutations (**Supplementary Figure 4**).

**Figure 3:**
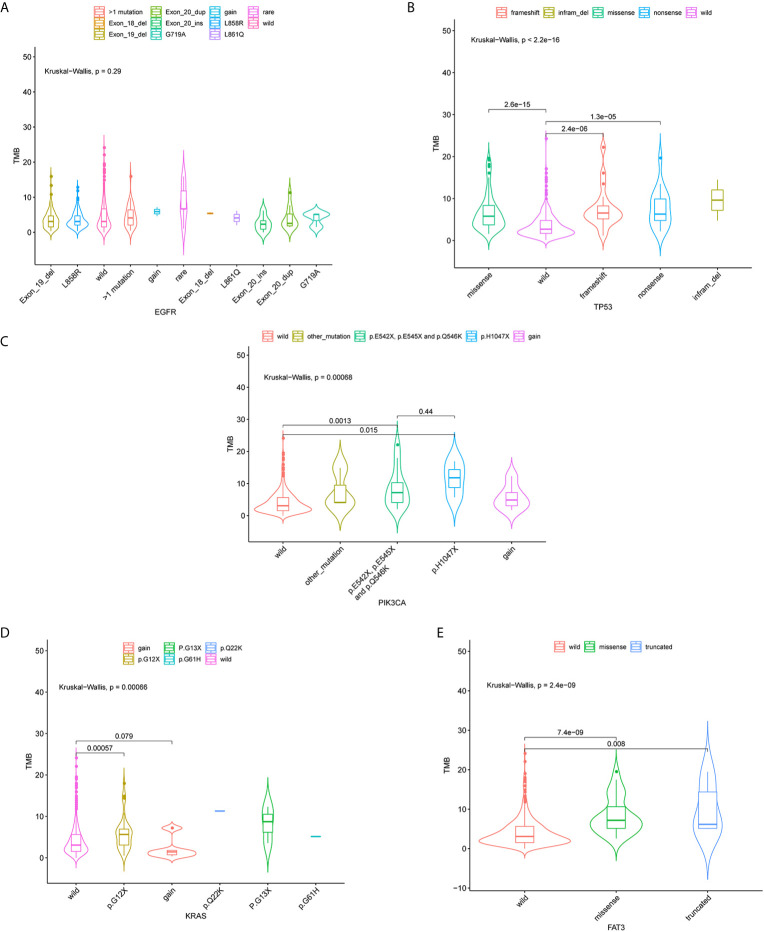
Violin plots of EGFR, TP53, PIK3CA, KRAS gene mutation types and the distribution of tumor mutation burden (TMB). Correlation between TMB and EGFR **(A)**, TP53 **(B)**, PIK3CA **(C)**, KRAS **(D)** and FAT3 **(E)** mutations.

Associations of four mutated genes with high frequency (*TP53*, *EGFR*, *KRAS*, and *FAT3*) and TMB were further investigated. 124 patients (24.85%) harbored *EGFR* L858R mutation, and 93 had *EGFR* exon 19 deletion (**Figure 3A**). No correlation of *EGFR* mutation status with TMB distribution was observed (P = 0.29) (**Figure 3A**). *TP53* mutations (missense, nonsense, and frameshift mutations) were significantly associated with TMB distribution (P  <  0.001) (**Figure 3B**). TMB was also significantly affected by three mutation types in *PIK3CA* genes, including p.E542X, p.E545X, and p.Q546K (P < 0.05) (**Figure 3C**). There was a significant correlation between TMB distribution and *KRAS* P.G12X (P  <  0.05) (**Figure 3D**). Besides, missense and truncated *FAT3* mutations were significantly related to TMB (P < 0.05) (**Figure 3E**).

### Constructing Tumor Mutation Burden Prediction Model

Based on the above clinical and genetic results, we hypothesized whether combination of clinical and genetic features could predict TMB status. Therefore, we trained a multivariable logistic regression model that included clinical parameters, age, histology, clinical stage (TNM) as well as genetic factors (*TP53*, *FAT3*, *APC*, *EPHA3*, TERT, *LRRK2*, RB1, *PTPRD*, *STK11*, and *NF1*).

**Figure 4:**
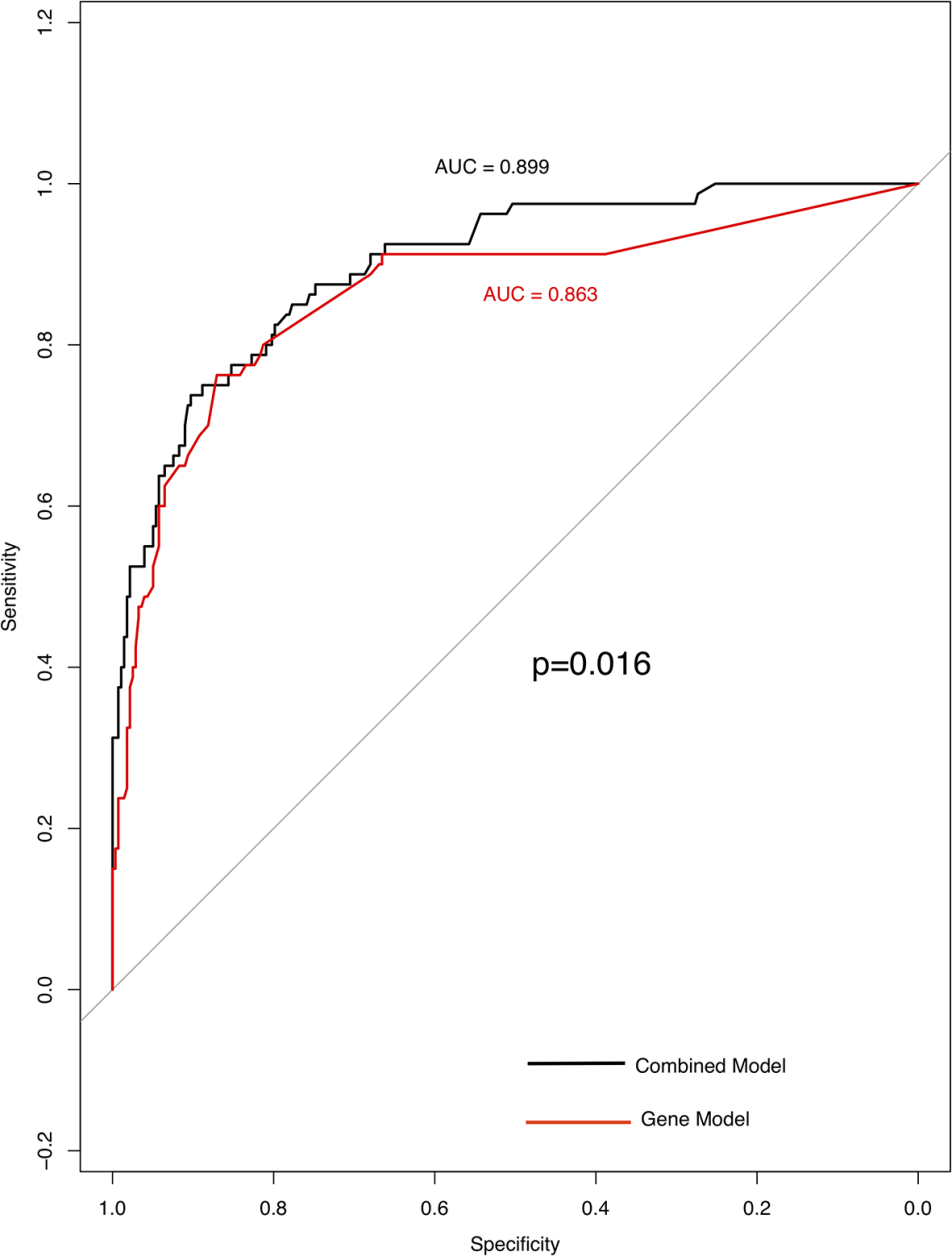
High specificity of genes and clinical model for predicting TMB status. ROC curve analysis was used to determine the sensitivity and specificity of the two models. The black curve is the combined model; the area under the ROC curve is 0.899(95% confidence interval: 0.861–0.938). Curve in red is gene model; the area under the ROC curve is 0.863 (95% confidence interval: 0.811–0.916).

Three factors (histology, Stage and *TP53*) were extremely powerful predictors for TMB through multivariate analysis (p < 0.001) (**Supplementary Table 1**). Other factors like *FAT3*, *APC*, *PTPRD* (P = 0.01), lymph-node metastasis, *EPHA3*, TERT, and *STK11* (P = 0.05) that have been found to be related to TMB distribution (**Supplementary Table 2**). Using TMB =6.15 muts/Mb, the prediction model achieved a sensitivity of 73.8% and a specificity of 90.3%; the AUC (area under the ROC curve) was 0.899 (95% confidence interval, 0.861–0.938) indicating its potential for reliably identifying patients with greater TMB. After removing histological parameters, the AUC (area under the ROC curve) of these factors was 0.863 with a sensitivity of 76.3% and a specificity of 87.1% (**Figure 4**).

## Discussion

TMB, PD-1/L-1 expression are used to select patients who may benefit from immunotherapy (13). TMB is an emerging predictive marker of immune checkpoint blockade response (4) as well as prognosis for patients with NSCLC (14). In particular, it is important to accurately predict the benefit of immunotherapy based on TMB status. Also, it is reported that high TMB was associated with a better prognosis in patients with resected NSCLC (15). Our integrated histologic and genomic model is an important step toward addressing this unmet need.

In this analysis, we evaluated the association between TMB and clinical characteristics in patients with early-stage NSCLC. In the univariate analysis, despite being significantly associated with sex, our results further found that TMB was correlated with several clinicopathological features, like histological subtype, LVI, pathological subtype, para-bronchial lymph nodes, lymph node metastasis as well as tumor size. A recent study also explored the correlation of TMB and clinical characteristics in early-stage squamous cell lung carcinoma; however, no significant association was observed between TMB and age, gender, smoking history, stage (16). Another study assessed associations between clinical and TMB in resected NSCLC and identified that histological type, gender, and smoking status were associated with higher TMB (17). These inconsistent findings may be due to differences in ethnicity and pathologic types of the cohorts. In addition, the differences in panels used for TMB evaluation also have a significant impact on the results. TMB was initially detected using whole exon sequencing, but a growing number of clinical trials are now using commercial panel sequencing to detect TMB. There is no uniform standard for TMB calculation method and threshold determination. Moreover, TMB varies greatly among different cancers and even different pathological subtypes. These are challenges that need to be overcome before further application of TMB (18–20).

In LUAD, carcinoma *in situ*, invasive and microinvasive cancers have different cell growth patterns and stages, which in turn affect the patient’s treatment and prognosis (21). In stage I LUADs, the micropapillary component was significantly associated with nodal micro-metastasis of tumor cells and may be a manifestation of aggressive behavior (22). TMB discrepancy was observed among LUAD with various components. Remarkably, it is critical to determine the heterogeneity of LUAD components (histological subtype) by genetic profile. Solid predominant LUADs were more likely to harbor *KRAS* mutations than are other predominant subtypes (23). The solid predominant subtype of tumor has been found to correlate remarkably with an inflamed phenotype characterized by a high proportion of PD-L1/CD8+TILs and active cytotoxic immune profiling and that increased tumor immunogenicity from a high TMB (24). The alterations of *EGFR*, *KRAS*, and *BRAF* genes proved to be more frequent in micropapillary LUAD (24, 25). The studies have suggested that the molecular pathogenesis of micropapillary component may differ from other types of LUAD (26).

Pre-invasive LUAD displayed distinct mutation profiles. *In situ* and micro-invasive LUAD showed higher prevalence of driver mutations, for example, *EGFR* mutations and *ALK* fusion. Thus, compared with invasive LUAD, *in situ* and micro-invasive LUAD had lower TMB, which are concordant with variant distribution of driver gene mutations in these two histologic subtypes. LUSC (27) and LVI (28) were previously found to have higher TMB, which was similar to the results of our study. Beside, LVI has been linked to an increase in immune cell infiltration (28). Our result, together with other reported data may provide a TMB related immune activation hypothesis. Patients with different tumor stages exhibited distinct clinical behaviors (29). Para-bronchial lymph nodes is associated with a poorer prognosis (21, 30). Thus, we inferred that these factors may affect immunogenicity through immune microenvironment and molecular profile. Neuro-invasive and stage-M did not affect TMB distribution, contrary to our expectation. The specific mechanism needs to be elucidated.

On the genomic level, the results showed that fifteen genes (*TP53*, *PIK3CA*, *KRAS*, *EPHA3*, *TSHZ3*, *FAT3*, *NAV3*, *KEAP1*, *NFE2L2*, *PTPRD*, *LRRK2*, *STK11*, *NF1*, *KMT2D*, *GRIN2A*) were significantly associated with TMB-H; *EGFR* was associated with TMB-L. Among these high-TMB-related genes, recent studies have shown that *TP53*-mutated tumors showed prominently increased somatic mutation burden compared with other mutant groups (*KRAS*, *EGFR*, *STK11*); and patients with *TP53* or *KRAS* mutations showed remarkable clinical benefit to PD-1 inhibitors (31, 32). *PIK3CA* and *KRAS* are mainly involved in the PI3K signaling pathway, which is one of the most important signal transduction pathways in the development of LUADs (33). In particular, four activated mutations of *PIK3CA* (p.E542X, p.E545X and p.Q546K) were found to have significant effect on TMB-H. *PIK3CA* gene mutations in the helical domain were correlated with TMB-H and poor prognosis in metastatic breast carcinomas with late-line therapies (34). Studies in lung cancer suggested that *PIK3CA* amplification was associated with higher TMB (8). Moreover, *KRAS* G12 mutations also correlated with high TMB group. As reported, NSCLC patients with *KRAS* G12 mutations showed an increased proportion of PD-L1+/CD8+TILs (35). These genes stimulated PTEN/*PIK3CA*/AKT pathway, which in turn would lead to increased proliferation of tumor cells (32). Accelerated cell cycle may accumulate somatic mutations putatively resulting in elevated TMB. *KEAP1*-*NFE2L2* plays a significant role in the dysregulation of oxidative stress pathway in lung cancer (36). Oxidative stress can lead to mutagenic DNA damage in the form of oxidative base modifications and the induction of DSB (DNA Double-Strand Break) which promotes mutations (8, 37). *KEAP1* mutation was significantly associated with lower CD8+TIL density which may be associated with shorter survival in LUAD patients receiving immnotherapy (38). It means that oxidative stress is a parallel mechanism of high-TMB. *EGFR*, *KRAS*, *TP53*, and *STK11*, also reported in a recent study, showed a correlation with tumor antigenicity and PD-L1 expression (8, 36, 39, 40). Of note, *GRIN2A* regulates excitatory neurotransmission in the brain (36) and has scarcely been reported in NSCLC. It is necessary to further obtain a deeper understanding of its mechanism and further applications. Our data also confirmed the association between *EGFR* mutations and TMB-L, which have been reported previously (41). Considering these findings, we speculate that tumor with high TMB-related-gene mutations may lead to the destruction of immune cells including CD8+TILs and DSB/DDR level, resulting in the increase of somatic mutations of tumor cells.

>Thus, we trained a multivariable logistic regression model predicting TMB category with 6.15 mutations/Mb as the cut-off value utilizing five clinical features (age, histology, T, N, M) and 10 genes (*TP53*, *FAT3*, *APC*, *EPHA3*, TERT, *LRRK2*, RB1, *PTPRD*, *STK11* and *NF1*). By comparing sensitivity and specificity, the results of two predictive model for TMB (histologic + genomics, genomics) confirmed that histologic features made a strong contribution to the integrated model for TMB prediction. There is also a small sample study which found that integrating multiple factors helps accurate prediction of TMB (37) although the ROC curves of the two studied models are close (0.89). Our study selected fewer histologic parameters without involving radiologic parameters. Another study showed that 56-gene panel could be used as a screening method for patients with low TMB. Compared with the panel, our prediction model has fewer genetic parameters, but achieved comparative efficiency (41). Therefore, this model may be better used to screen TMB-L or TMB-H status in early-stage NSCLC patients.

## Conclusion

Overall, comprehensive clinical and genomic information can effectively evaluate TMB-high or low status. Our results showed that an integrated prediction model combining histology and genomic parameters significantly improved the accuracy of TMB prediction. However, whether this integrated model plays a key role in predicting the clinical outcome to immunotherapy and prognosis, still needs further investigation.

## Data Availability Statement

The datasets presented in this study can be found in online repositories. The names of the repository/repositories and accession number can be found below: China National GeneBank DataBase (CNGBdb) https://db.cngb.org/. Accession number CNP0001479.

## Ethics Statement

The studies involving human participants were reviewed and approved by Institutional Review Board of the First Affiliated Hospital of Guangzhou Medical University. The patients/participants provided their written informed consent to participate in this study. The animal study was reviewed and approved by Institutional Review Board of the First Affiliated Hospital of Guangzhou Medical University.

## Author Contributions

JH, YQ, CZ, and LL conceptualized and designed the study. KW provided administrative support. YY and HC provided the study materials or patients. QD, YL, and WJ collected and assembled the data. WL and DS analyzed and interpreted the data. All authors contributed to the article and approved the submitted version.

## Funding

This study is funded by the Foundation and Applied Basic Research Fund of Guangdong Province (02020A1515011293) and the National Natural Science Foundation of China (Grant No. 81772486).

## Conflict of Interest

CZ, WL, DS, KW and WJ are employees of BGI Genomics that produces the panel test used in this study.

The remaining authors declare that the research was conducted in the absence of any commercial or financial relationships that could be construed as a potential conflict of interest.

## Supplementary Material

Supplementary Figure 1 The distribution of TMB.

Supplementary Figure 2 Driver mutation status and association with TMB.

Supplementary Figure 3 The distribution of high-frequency genes in the TMB-H and TMB-L groups.

Supplementary Figure 4 ICI related genes and association with TMB.

Supplementary Table 1 Correlation between TMB and high frequently mutated genes.

Supplementary Table 2 Gene and combined model for predicting TMB in NSCLC.
