# Supplementary figures and images for: Integrating Histologic and Genomic Characteristics to Predict Tumor Mutation Burden of Early-Stage Non-Small-Cell Lung Cancer
Source: Front Oncol. 2021 Apr 30;10:608989. doi: 10.3389/fonc.2020.608989 (PMC8121003; doi:10.3389/fonc.2020.608989)

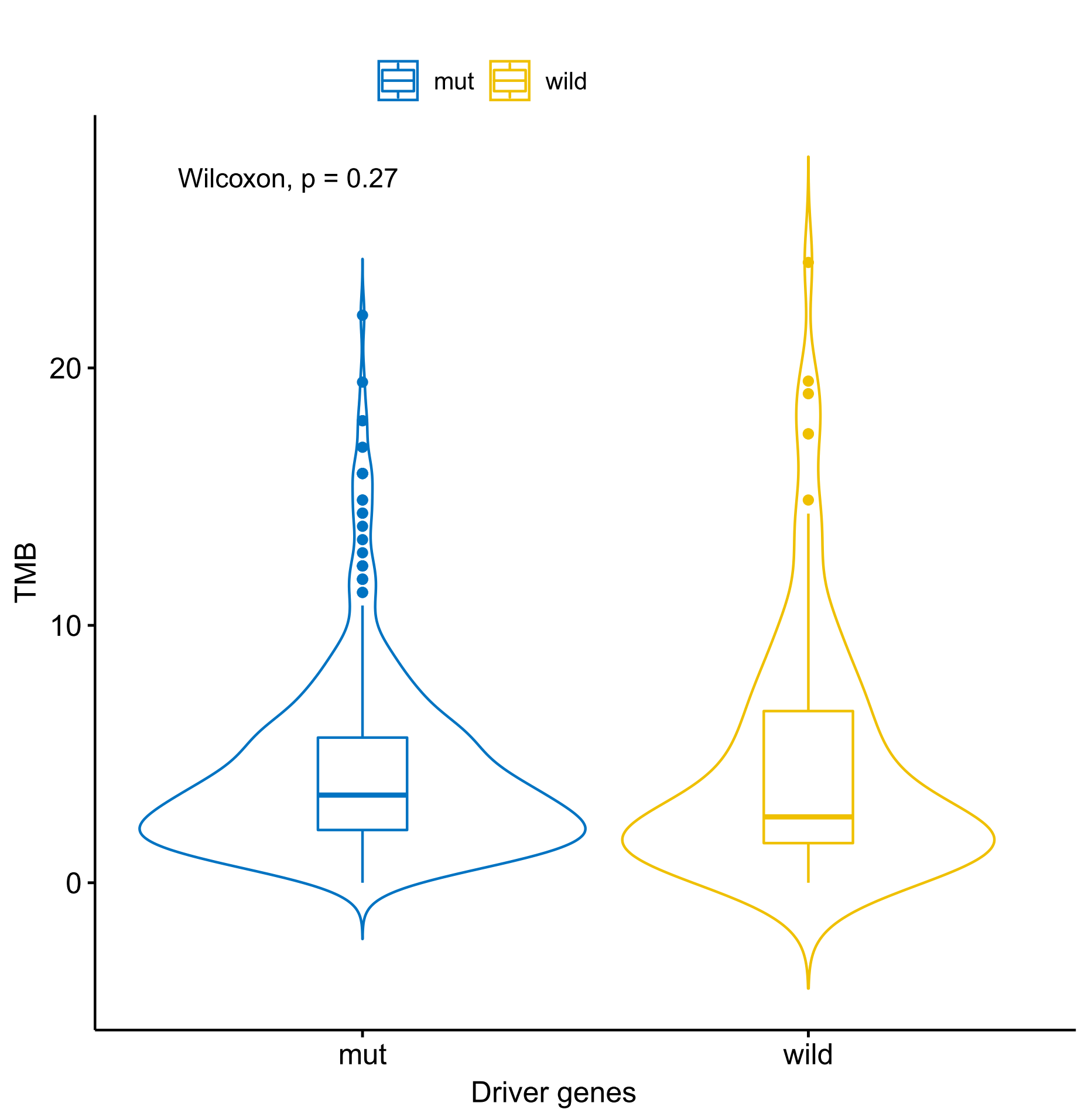

Supplement: Supplementary Figure 2 — Driver mutation status and association with TMB. [file Image_2.tif]

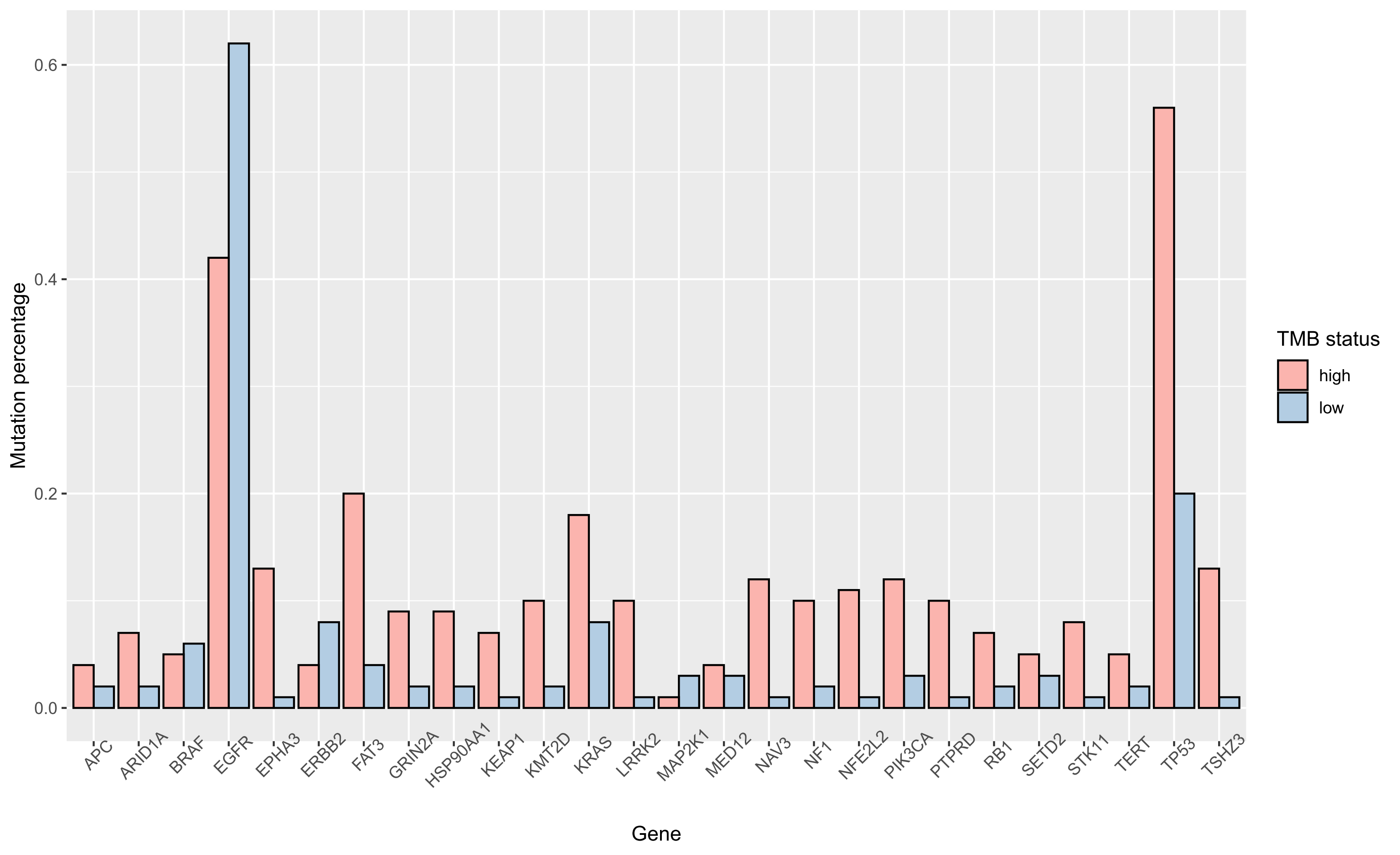

Supplement: Supplementary Figure 3 — The distribution of high-frequency genes in the TMB-H and TMB-L groups. [file Image_3.tif]

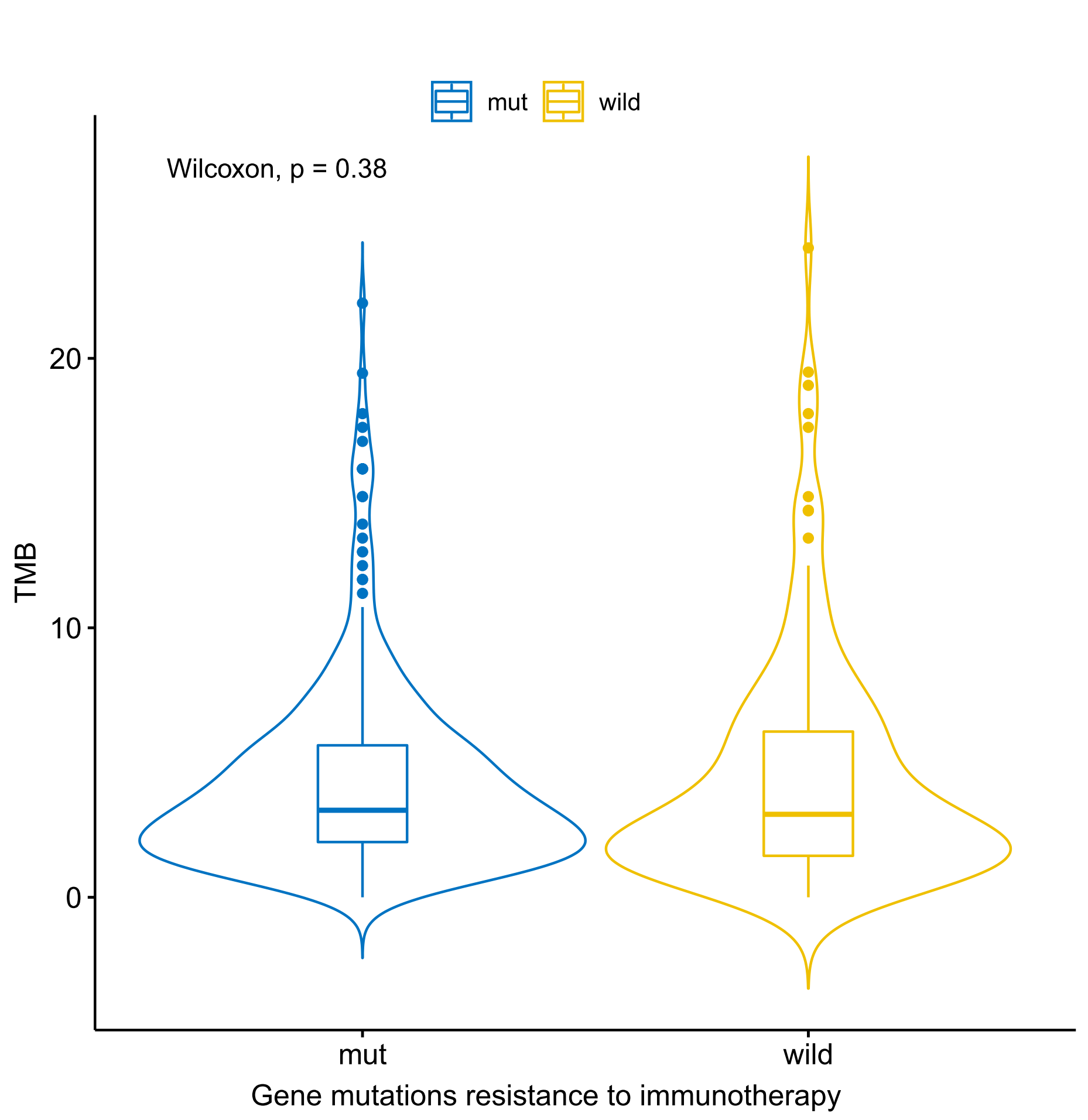

Supplement: Supplementary Figure 4 — ICI related genes and association with TMB. [file Image_4.tif]
