# Supplementary material for: Integrating Histologic and Genomic Characteristics to Predict Tumor Mutation Burden of Early-Stage Non-Small-Cell Lung Cancer
Source: Front Oncol. 2021 Apr 30;10:608989. doi: 10.3389/fonc.2020.608989 (PMC8121003; doi:10.3389/fonc.2020.608989)
Supplement: Supplementary Table 1 — Correlation between TMB and high frequently mutated genes. [file Table_1.docx]

Table S1. Multivariate analysis of TMB using genes

| Gene | TMB low | TMB high | p |
| --- | --- | --- | --- |
| APC | 0.02 | 0.04 | 0.425 |
| ARID1A | 0.02 | 0.07 | 0.022 |
| BRAF | 0.06 | 0.05 | 0.888 |
| EGFR | 0.62 | 0.42 | <0.001 |
| EPHA3 | 0.01 | 0.13 | <0.001 |
| ERBB2 | 0.08 | 0.04 | 0.246 |
| FAT3 | 0.04 | 0.2 | <0.001 |
| GRIN2A | 0.02 | 0.09 | 0.002 |
| HSP90AA1 | 0.02 | 0.09 | 0.002 |
| KEAP1 | 0.01 | 0.07 | 0.001 |
| KMT2D | 0.02 | 0.1 | <0.001 |
| KRAS | 0.08 | 0.18 | 0.005 |
| LRRK2 | 0.01 | 0.1 | <0.001 |
| MAP2K1 | 0.03 | 0.01 | 0.443 |
| MED12 | 0.03 | 0.04 | 0.851 |
| NAV3 | 0.01 | 0.12 | <0.001 |
| NF1 | 0.02 | 0.1 | <0.001 |
| NFE2L2 | 0.01 | 0.11 | <0.001 |
| PIK3CA | 0.03 | 0.12 | <0.001 |
| PTPRD | 0.01 | 0.1 | <0.001 |
| RB1 | 0.02 | 0.07 | 0.022 |
| SETD2 | 0.03 | 0.05 | 0.5 |
| STK11 | 0.01 | 0.08 | <0.001 |
| TERT | 0.02 | 0.05 | 0.184 |
| TP53 | 0.2 | 0.56 | <0.001 |
| TSHZ3 | 0.01 | 0.13 | <0.001 |
